# Accounting for Limited Detection Efficiency and Localization Precision in Cluster Analysis in Single Molecule Localization Microscopy

**DOI:** 10.1371/journal.pone.0118767

**Published:** 2015-03-20

**Authors:** Arun Shivanandan, Jayakrishnan Unnikrishnan, Aleksandra Radenovic

**Affiliations:** 1 Laboratory of Nanoscale Biology, Institute of Bioengineering, Ecole Polytechnique Federale de Lausanne (EPFL), Lausanne, Switzerland; 2 Audiovisual Communications Laboratory, School of Computer and Communication Sciences, Ecole Polytechnique Federale de Lausanne (EPFL), Lausanne, Switzerland; Wake Forest University School of Medicine, UNITED STATES

## Abstract

Single Molecule Localization Microscopy techniques like PhotoActivated Localization Microscopy, with their sub-diffraction limit spatial resolution, have been popularly used to characterize the spatial organization of membrane proteins, by means of quantitative cluster analysis. However, such quantitative studies remain challenged by the techniques’ inherent sources of errors such as a limited detection efficiency of less than 60%, due to incomplete photo-conversion, and a limited localization precision in the range of 10 – 30nm, varying across the detected molecules, mainly depending on the number of photons collected from each. We provide analytical methods to estimate the effect of these errors in cluster analysis and to correct for them. These methods, based on the Ripley’s *L(r) – r* or Pair Correlation Function popularly used by the community, can facilitate potentially breakthrough results in quantitative biology by providing a more accurate and precise quantification of protein spatial organization.

## Introduction

The spatial organization of most membrane proteins as sub-micrometer spatial clusters might be a key property affecting their functionality [1, 2, 3]. The characteristics of these microdomains, such as the number of proteins per cluster, cluster size and density, are heterogeneous in general, depending on the specific cell type, protein, lipid, cell cycle and environmental conditions. The possible mechanisms of cluster formation include compartmentalization due to enrichment in lipid rafts [4], protein-protein interactions [1] and physical barriers created by actin cytoskeleton [5]. Various biological functions, for example signalling [2, 3], might be facilitated by clustering. Accurate and precise imaging and quantitative characterization of the spatial microdomain parameters are important tools that can aid these studies.

The membrane proteins can be imaged at unprecedented length-scales, and with high specificity and contrast and in their natural environment, using single molecule localization microscopic (SMLM) techniques like PhotoActivated Localization Microscopy(PALM) [6, 7]. Other SMLM techniques like STochastic Optical Reconstruction Microscopy (STORM) [8] also are promising, although, due to the high specificity provided by PALM originating from the genetic tagging of the protein under study with the fluorescent label, and the relatively lower blinking rate of the Photo-Activable Flourescent Proteins (PA-FPs) and the resulting reduction in the number of repeated localizations of the same fluorophore molecule, we focus on the application of PALM for quantitative analysis, while noting that the methods provided might be applicable to STORM as well. The technique has been popularly used for quantitative analysis of spatial organization of membrane proteins [9, 10, 11, 12, 13]. Such studies typically also involve the use of cluster analysis methods for quantification.

However, the quantitative analysis of membrane organization with PALM has several sources of errors that can significantly limit its utility [10, 14, 15, 16, 17, 18, 20, 21]. The most critical sources of these errors are: a) multiple localizations of the same label molecule due to fluorophore blinking [18, 19] b) inability to image all the label molecules, due to a limited detection efficiency of typically 40%–60%, resulting from inherent errors like imperfect fluorescent protein folding, maturation and photo-conversion [16, 17] c) localization uncertainty, or the error in the estimation of the position of the label molecule due to the limited number of photons collected from them, commonly represented by the localization precision in the range of 10–30nm [20, 21]. All of these can possibly cause significant variations in quantitative analysis([14, 15, 16, 17, 18, 19], also S1 Fig.). Additionally, the finite label and linker size, stage drift and effects due to fixed dipole orientation of the emitter molecules can affect the accuracy and precision of localizations [15, 22, 23]. These sources of errors are applicable to other SMLM techniques like STORM, though in different magnitudes.

Several methods have been proposed to account for the multiple appearances due to blinking [10, 18, 19, 24]. However, the other two sources of error, the subsampling due to limited detection efficiency, and the effect of localization uncertainty on measured cluster properties, remain problematic. A method has been proposed to account for localization uncertainty in analysis [25], however, in the context of very small clusters (typically upto tetramers) and low molecular density, and based on simulations. Currently, several of the cluster analysis studies based on PALM perform the analysis and comparisons of estimated cluster properties without accounting for these satisfactorily, possibly resulting in inaccurate results (S1 Fig.).

In this work, we focus on these two problems. Based on a well-known result from spatial statistics, we demonstrate that the commonly used tools for cluster analysis such as Ripley’s *K* function, *L*(*r*) − *r* function and the related Pair Correlation Function (PCF) are invariant to random subsampling, and hence, within limits, are unaffected by limited detection efficiency. We then provide a method to estimate true fluorophore locations from the localizations appearing in clusters, and to correct for errors due to localization uncertainty. These estimated true locations can be used to estimate the true Ripley’s *L*(*r*) − *r* function or the PCF, and thus provide better estimates of true cluster parameters.

Before proceeding further, we note that, while the methods discussed in the paper are focussed on the *L*(*r*) − *r* function, they should be applicable to the PCF as well (Methods). We also note that the result on invariance to random subsampling property applies to the bivariate versions of the *K*, *L*(*r*) − *r* and PCF functions (called Cross Correlation Function or CCF), and hence on related co-localization measures [10].

## Results

### Accounting for limited detection efficiency

We consider the situation when the phenomenon of limited detection efficiency in a PALM experiment can be modelled as a case of random, independent, homogeneous subsampling of the label molecules present in the sample. Such a model is valid if the effect of limited detection efficiency, i.e whether a flourophore that is present in the field of view is imaged or not, is independent of its spatial location. In the case of spatial homogeneity in illumination and environmental conditions such as pH, and in the absence of inter-fluorophore molecule interactions, this assumption is reasonable.

**Fig 1:**
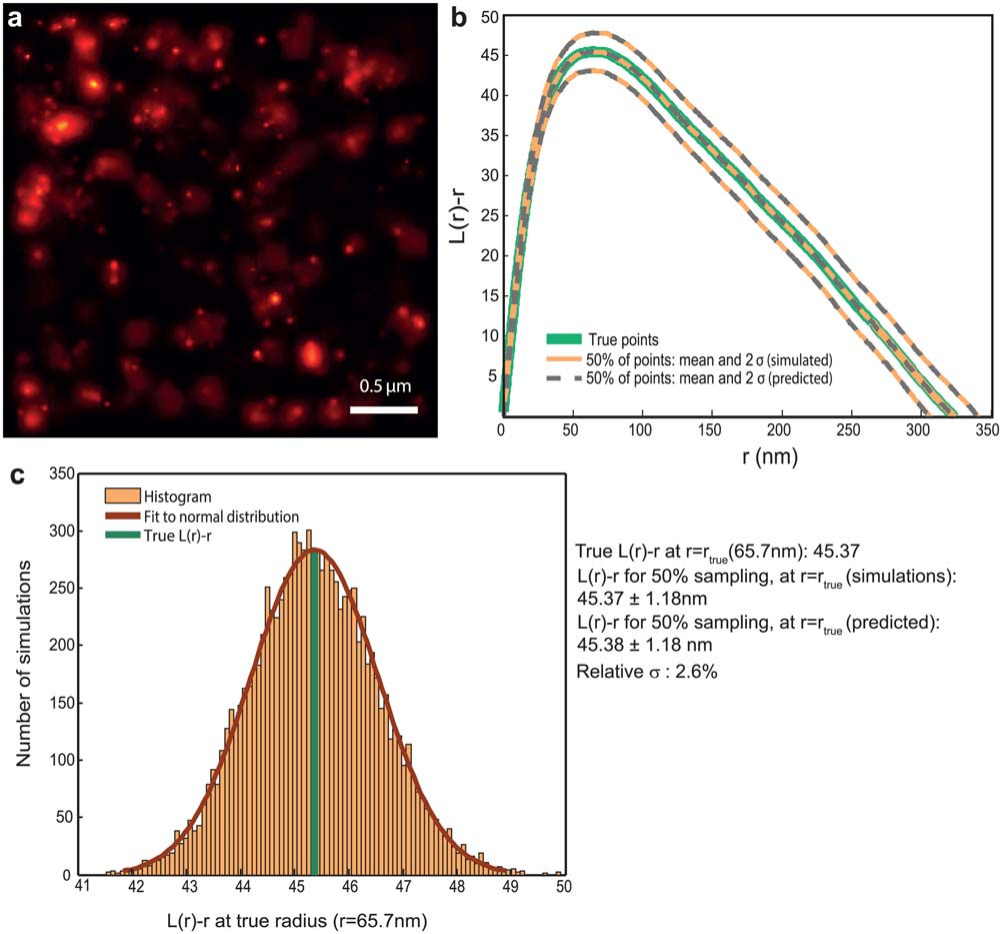
Ripley’s *L*(*r*) − *r* function is invariant to random subsampling. (a) Probability map representation of a PALM image of *β*2–adrenergic receptor molecules labeled with mEos2 on the plasma membrane of HeLa cells, post agonist addition. Density: 650 molecules/*μm* ^−2^. (b) *L*(*r*) − *r* functions for the true and subsampled points, estimates for the latter obtained from both simulations and the analytical method presented. Continuous green: Ripley *L*(*r*) − *r* function *L*_*true*_(*r*) − *r* corresponding to the points in (a). Orange: mean and 2*σ* bounds of *L*(*r*) − *r* functions corresponding to 10000 realizations of random sampling 50% of the points in (a). Broken lines: the mean and 2*σ* bounds corresponding to 50% subsampling, predicted by the analytical method presented. It can be seen that the mean values obtained from both simulations and analytical method coincide with *L*_*true*_(*r*) − *r*, and that the 2*σ* curves obtained from the simulations and the analytical method coincide. (c) Histogram of *L*(*r*) − *r* of the subsampled realizations at *r* = *r*_*true*_, where *r* = *r*_*true*_ is the cluster radius corresponding to the maxima of the *L*_*true*_(*r*) − *r* function. It can be seen that it follows a normal distribution, with the fit parameters similar to that obtained from the analytical method. *r*_*true*_ is also plotted (dark green). The relative standard deviation (*σ*/*μ*, i.e, $\frac{\sigma_{subsampled}}{L_{true}(r)-r}$) at *r* = *r*_*true*_ is 2.6% for 50% subsampling.

We then use the result from spatial statistics that analytically shows that the *L*(*r*) − *r* function is invariant to random subsampling of the underlying point pattern [26] (Methods, Fig. 1). The result arises from the fact that, in the event of subsampling, both the numerator and the denominator of these measures gets scaled similarly and hence they cancel out, on average(details in Methods).

Due to the stochasticity involved in random subsampling, the *L*(*r*) − *r* function corresponding to the subsampled point pattern (i.e localizations corresponding to the detected molecules), called *L*_*subsampled*_(*r*) − *r*, will also be a stochastic quantity (and a PALM experiment provides one realization of the subsampling). For a stationary point pattern, the *L*_*subsampled*_(*r*) − *r* at any point *r* can be approximately modeled as a random variable with a normal distribution centered around a mean value; this mean *L*_*subsampled*_(*r*) − *r* provides an unbiased estimate for *L*_*true*_(*r*) − *r* as per the result above. We provide an analytical method to exactly compute the standard deviation of the *K* distribution of the subsampled points at each point *r*, and approximately compute that of *L*(*r*) − *r* (called *σ*_*subsampling*_), given the true set of points (Fig. 1, Methods, S2 Fig.) and the sampling ratio.

We empirically characterized the *σ*_*subsampling*_ corresponding to various cluster and subsampling conditions, by means of simulated points (S3 Fig.). Simulated clustered point patterns were created for varying cluster density (total number of clustered points in the area under analysis, conditions tested: 10, 100 and 1000 *μm* ^−2^), cluster size (*σ* of Gaussian, 10,30,50 and 100) and number of points per cluster (10 and 100). The effect of sampling ratios of 20%, 40%, 60% and 80% were tested. In all the clustering conditions tested, the ratio of *σ*_*subsampling*_ to the *L*_*true*_(*r*) − *r* at *r* = *r*_*true*_, called relative standard deviation, *r*_*true*_ corresponding to the point *r* in which *L*_*true*_(*r*) − *r* is maximum, remained less than .25 for 60% sampling, with most conditions having a value less than .15. For several of the cluster conditions, the ratio obtained was less than .1 even for 20% sampling. The characterization for smaller clusters (dimers and tetramers) can be found in S4 Fig., with similar results (ratio less than .15 for 60% sampling).

**Fig 2:**
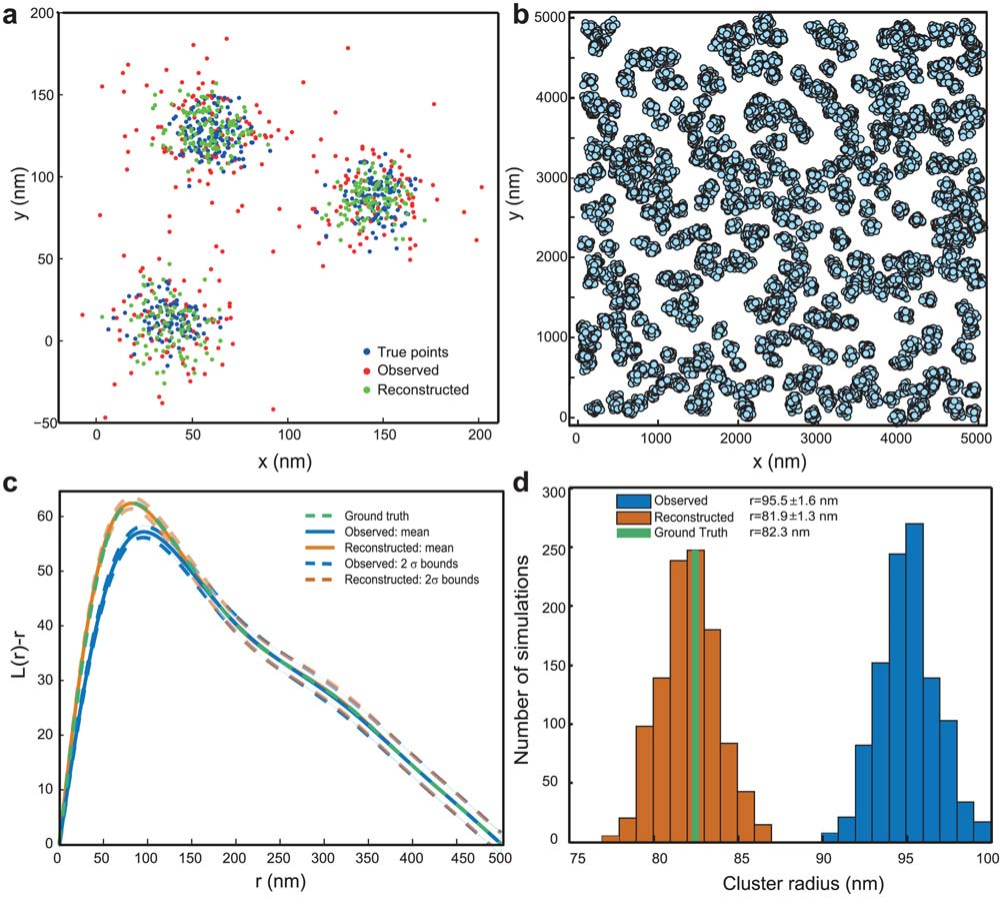
Reconstruction of true point locations from noisy observations. (a) Demonstrative example of reconstruction method. The true (blue), observed (red) and reconstructed (green) locations, in the case of 3 Gaussian clusters with average cluster standard deviation of 10nm observed with an average localization uncertainty (*σ*) of 17nm. It can be seen that the observed clusters (average *σ* ≈ 22nm) are enlarged with respect to the true clusters, and the reconstructed clusters are closer to the true ones. (b, c, d): Example of estimation of true cluster properties from simulations of clustered points with limited detection efficiency and localization errors added. (b) Ground truth: simulated membrane protein cluster. Each cluster is assumed to be Gaussian shaped (*σ* = 30nm), with 40 points on average. The overall density in the area of analysis is 760 molecules/*μm* ^2^. The true points are observed with a limited detection efficiency of 50% and a mean localization precision of 18nm. (c) Ripley *L*(*r*) − *r* functions corresponding to true points (green), mean and 2*σ* envelopes for 1000 simulations of observed data (cyan) and reconstructions from them (orange). (d) Histograms of cluster radius estimated from the maxima of *L*(*r*) − *r* curves in (c), for the observed data (cyan) and the reconstructions (orange), with the value corresponding to the ground-truth (green). It can be seen that the reconstructed values (81.9 ± 1.3 nm) are very close to the ground-truth (82.3 nm), compared to the observed values (95.5 ± 1.6nm).

Since the *r* value corresponding to the maxima of *L*(*r*) − *r* function can provide an estimate of the cluster radius, the invariance property of *L*(*r*) − *r* could also be useful for its accurate estimation (Fig. 2).

It should be noted that in this work, subsampling is assumed to be spatially homogeneous. Apart from assuming that the limited detection efficiency due to physical phenomena is spatially homogeneous, it also assumes that the errors during the computation of localization estimation are also spatially homogeneous; e.g., the phenomena of higher number of missed localizations in denser regions due to overlapping Point Spread Functions is minimal. It is also important for accurate and precise estimation of the *L*(*r*) − *r* functions that the effect of sampling errors is not significant. Therefore, in the case of very few clustered points in the area of analysis, it is important to validate the applicability by means of simulations.

### Estimating true point locations in the presence of localization error

We propose a method that estimates the true fluorophore locations from imaged localizations that appear in clusters, accounting for localization uncertainty. Spatial clusters tend to appear more dispersed and hence enlarged, when the localizations involve measurement error (uncertainty). Thus naïve estimates of Ripley’s *L*(*r*) − *r* function that ignore localization uncertainties are biased even when the uncertainties are unbiased (S1 Fig.). However, the amount of enlargement for a given cluster can be predicted as a function of the precisions of all localizations. We show that if the precisions of all localizations are known, the true position corresponding to each localization in an observed cluster can be estimated (Methods, Fig. 2). A model for localization precision based on the photon count can generally be identified [20, 21] and can be used for this estimation, even though such models ignore other sources of estimation errors (finite label and linker size and orientation, stage drift, fixed emission dipole orientation). Hence such models should be considered as a lower bound for localization error.

These position estimates, obtained after correcting for the enlargement of the cluster due to localization uncertainty, can be used for more accurate cluster analysis, whether by means of exploratory tools like the *L*(*r*) − *r* or PCF functions presented in this paper, or through clustering by means of algorithms like DBSCAN [27] followed by estimation of each cluster’s properties [14].

In the case of using tools such as *K*, *L*(*r*) − *r* or PCF, the property of invariance to random subsampling presented in the paper can correct for limited detection efficiency as well (Fig. 2).

**Fig 3:**
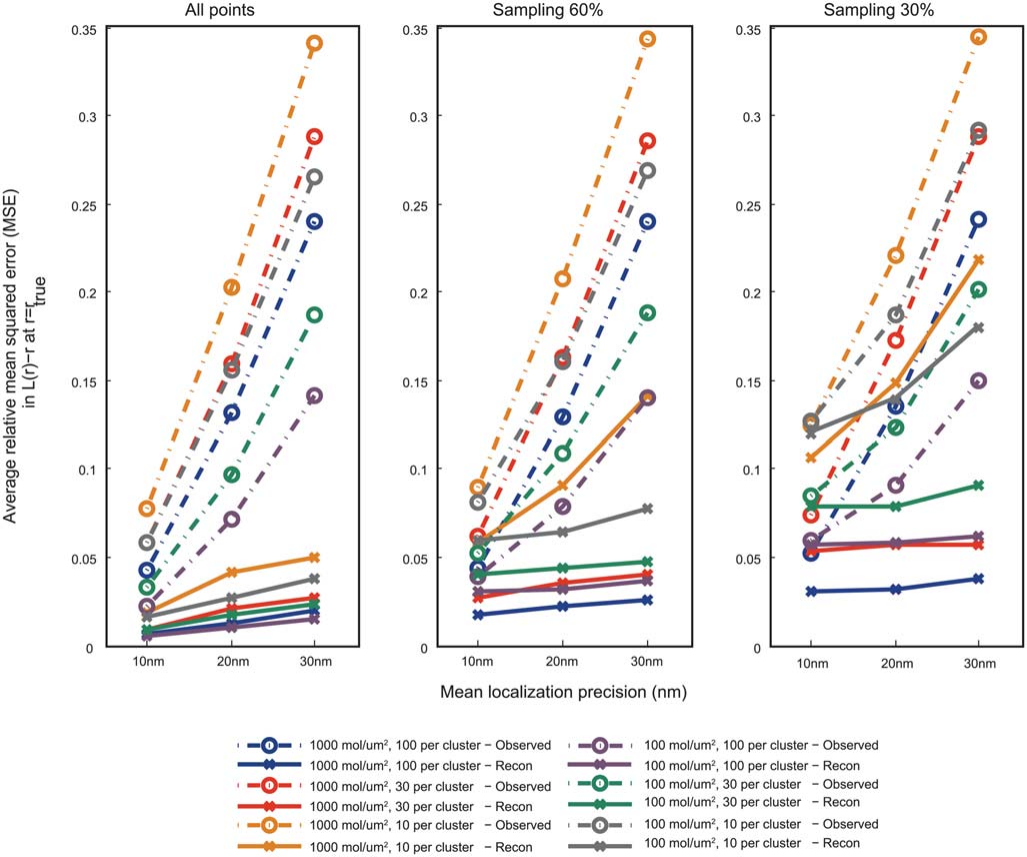
Characterization of error in *L*(*r*) − *r* for observed and reconstructed points to that corresponding to true points, at *r* = *r*_*true*_, based on simulated point patterns. The simulations were done as described in Methods. Cluster size (*σ*) of true point pattern: 30nm. The observed points were obtained from true points by applying localization errors with mean localization precision 10, 20 and 30nm, and sampling rates of 30%, 60% and 100% (40 realizations per condition). The reconstructed points were obtained from these points by means of the methods presented in the paper. The mean squared error in *L*(*r*) − *r* between the ones corresponding to realizations of the observed/reconstructed points and the true points is computed at *r* = *r*_*true*_, the true cluster radius estimated from the maxima of *L*_*true*_(*r*) − *r*, and is then divided by *L*_*true*_(*r*) − *r* to make it relative. The relative MSE is averaged over 10 realizations of point patterns corresponding to the same cluster conditions. Broken lines: error rate corresponding to observed points, Solid lines: corresponding to reconstructed points. It can be seen that the reconstruction method provides significant reduction in error for sampling rates of 60% and 100% for all clustering conditions. The reduction in error remains significant even for 30% sampling, except for the case when the average number of points in a cluster is only 10, in which case also there is considerable improvement.

The methods are validated by simulations (Figs. 2, 3, S5 Fig.). For a simulated clustered point pattern with a density of 760 points *μm* ^−2^ and with a true cluster radius of 82.3nm, while observation with an average localization precision of 18nm and a subsampling of 50% provided a cluster radius of 95.5 ± 1.6 nm, the reconstruction and estimation with the presented method provided a radius of 81.9 ± 1.3 nm. Characterization of the accuracy and precision of the reconstruction method for different cluster and error conditions can be found in Fig. 3. Details of the simulations used in the paper can be found in Methods. It should be noted that the reconstruction method is crucially dependent on the clustering of the localization data into appropriate shapes. A discussion of sources of error in reconstruction, especially that due to errors in clustering, can be found in S1 Text. Example reconstruction by setting observed PALM data as true points, and involving the errors due to clustering, can be found in S5 Fig. An example application of the method on a previously published cluster analysis data [13] can be found in S6 Fig., where the *L*(*r*) − *r* curve corresponding to the reconstructed points gives a measure of the possible deviation from that of the observed ones, due to the effect of limited localization precision. As expected, the cluster radius estimated for the reconstructed points(97.5 nm) was found to be 11.5 nm lower than one for the observed points(109 nm) that neglects the influence of limited localization precision.

We also provide an analytical method to exactly compute the mean *L*(*r*) − *r* function corresponding to uncertain localizations, as a function of the true point locations and localization precisions of all points (Methods, S7 Fig.). For a given PALM experiment, this method and the method to compute the variance due to subsampling can be used along with simulations of true points to compute the limits of minimum detection efficiency and localization precision permissible for a given target accuracy.

## Discussion

It can be seen from the figures that we have successfully demonstrated the applicability of invariance to subsampling property to PALM data, and the estimation of the variance of subsampling and the reconstruction aspects of the method perform well, with the reconstruction method providing significant reductions in estimation error. The method is simple to implement, and an implementation in MATLAB is provided. The advantages of the method include the fact that it is based on Ripley’s *L*(*r*) − *r* function or Pair Correlation Function, methods that are very popular in the field. It can also be easily coupled with other methods that solve the problem of overcounting due to photoblinking, using models based on the PCF [10]. The invariance to random subsampling property applies also to co-localization analysis with two-color *L*(*r*) − *r* function or Cross Correlation Function, where the problem of limited detection efficiency involves the multiplicative propagation of errors. The reconstruction method can also be used independently of *L*(*r*) − *r* or PCF functions. The methods’ applicability is not limited to PALM or SMLM, the invariance property might as well be applicable to Electron Microscopy based analysis of membrane proteins, where the immunolabelling of the ultrastructure involves major subsampling. We believe the method, by providing solutions for several important sources of error in SMLM, has the potential to form an invaluable tool for the community.

## Methods

### Invariance of *L*(*r*) − *r* function and PCF to random subsampling

For a stationary spatial point pattern defined in two-dimensional space, Ripley’s *K*-function is defined [26, 28] as

$$\begin{array}{c} K\left(r\right)=\frac{1}{\lambda }E\left[M\left(r\right)\right] \end{array}$$ (1)

where *λ* is the spatial density (average number of points per unit area), and *M*(*r*) is the number of extra events within distance *r* of a randomly chosen event. Ripley’s *L* function is then defined as

$$\begin{array}{c} L\left(r\right)=\sqrt{\frac{K(r)}{\pi }}. \end{array}$$ (2)

For points that are distributed in space with complete spatial randomness, i.e. without forming any special organization such as clustering, $\text{E}[M(r)]=\pi \lambda r^{2}$, and hence *L*(*r*) − *r* = 0. In the case of clustered point pattern, *L*(*r*) − *r* > 0 and in the case of anti-clustered (regularity), *L*(*r*) − *r* < 0. In the case of clustering, the magnitude of *L*(*r*) − *r* gives a sense of the degree of clustering (‘cluster strength’) compared to a point pattern distributed uniformly at random with the same density, and can be used for comparisons in relative clustering between point patterns. The *r* value corresponding to the maxima of *L*(*r*) − *r* gives a measure of the average cluster radius. We note that other measures based on the *L*(*r*) − *r* function to estimate the cluster radius are available [29], and the methods proposed in this work are independent of the specific measure used. Pair Correlation Function (PCF, represented by *g*(*r*)) is a closely related measure, in that *K*(*r*) is the integral of 2*πrg*(*r*). We also note that the radius estimate obtained is an ensemble measure, and may not reflect the variability within the individual cluster radii of various clusters in the area of analysis.

A well-known result [26, 28] in spatial statistics states the following. When a process is *randomly subsampled* by a factor *α*, by means of independent Bernoulli trials with probability *α*, the intensity *λ*, gets reduced to *αλ*. Simultaneously E[*M*(*r*)] also gets multiplied by a factor *α* (see [26] for a proof), and hence *K*(*r*) remains invariant to random subsampling. Similar argument holds for Pair Correlation Function (PCF) [26, 28] and *L*(*r*) − *r*.

### Exact computation of variance of *K*-function due to random subsampling

In practice the *K*-function is estimated using the following expression

$$\begin{array}{c} K\left(r\right)=\frac{\sum_{i\neq j}I\{∥X_{i}-X_{j}∥\leq r\}/N}{N/A} \end{array}$$ (3)

where the numerator is an estimate for the expected value in Eq. (1) and the denominator is an estimate for the intensity *λ*. Here *N* is the total number of points in the area under analysis, *A* is the total area, and *X*_1_, *X*_2_, …, *X*_*N*_ are the positions of the points, represented as vectors in ℜ^2^, and I{E} is the indicator function of an event *E*, taking the value 1 if the event *E* occurs and 0 otherwise.

Suppose the point pattern is randomly subsampled with a probability *α*. Then the estimate of the *K* function obtained from the subsampled point pattern is given by

$$\begin{array}{c} K\left(r\right)=\frac{\sum_{i\neq j}B_{i}B_{j}I\{∥X_{i}-X_{j}∥\leq r\}A}{{\left(\sum_{i=1}^{N}B_{i}\right)}^{2}} \end{array}$$ (4)

where *B*_*i*_ are independent binary variables following a Bernoulli distribution with mean *α*. For a given set of positions {*X*_1_, *X*_2_, …, *X*_*N*_} and a given sampling probability *α*, we analytically compute expressions for the expected value and variance of *K*(*r*), by using the binomial theorem. The details of the derivation are given in S1 Text. The corresponding values in *L*(*r*) − *r* can be approximately computed from the *K*(*r*) function obtained.

### Exact computation of *K*-function in the presence of localization uncertainty

In the presence of measurement error (localization uncertainty), the observed position of a point is a perturbed version of its true position. Let $X_{i}^{\prime }$ denote the observed position of the *i*-th point whose true position was at *X*_*i*_. Thus $W_{i}=X_{i}^{\prime }-X_{i}$ is the corresponding measurement error. The *K*-function estimate obtained by using the observed positions is thus given by

$$\begin{array}{ccc} K^{'}\left(r\right) & = & \frac{\sum_{i\neq j}I\{∥X_{i}-X_{j}+W_{i}-W_{j}∥\leq r\}/N}{N/A} \end{array}$$ (5)

For a given set of true positions and given distribution of the observation errors, the expected value of *K* ^′^(*r*) can be calculated. A derivation of an analytical expression for this expected value, under an assumption of independent normal distributions for measurement errors, is given in the S1 Text.

### Estimating true locations of molecules and true *L*(*r*) − *r* function

In order to correct for measurement errors, we employ a technique to estimate the true locations of the points given the variance in their observed locations. We ignore clustering errors and separately estimate the locations of points within each cluster. Let $X_{1}^{\prime },X_{2}^{\prime },\ldots ,X_{K}^{\prime }$ be the observed locations of the points within a cluster containing *K* points. Further, let $X_{i}^{\prime }=(x_{i}^{\prime },y_{i}^{\prime })$ be the representation of the *i*-th point in terms of its *x* and *y* coordinates. We estimate the *x* coordinates and *y*-coordinates of each point separately. Here we describe only the procedure for estimating the *x*-coordinates. The *y*-coordinates are estimated similarly. We model the true x-coordinates of all points in the cluster as i.i.d. random variables with an unknown mean *μ*_*x*_ and variance $\sigma_{x}^{2}$. Let $\sigma_{x,i}^{2}$ denote the measurement error variance in the *x*-coordinate of the *i*-th point. We first estimate the *x*-coordinate of the cluster center as

$$\begin{array}{c} \bar{x}=\frac{1}{K}\sum_{i=1}^{K}x_{i}^{'}, \end{array}$$ (6)

and then compute an estimate of the mean error in the *x*-coordinates as

$$\begin{array}{c} {\bar{\sigma }}_{x}^{2}=\frac{1}{K}\sum_{i=1}^{K}\sigma_{x,i}^{2}. \end{array}$$ (7)

Using this we estimate the true spread in the *x*-coordinates of the cluster. Let

$$\begin{array}{c} {\overset{ˇ}{\sigma }}_{x}^{2}=\frac{1}{K-1}\sum_{i=1}^{K}{\left(x_{i}^{'}-\bar{x}\right)}^{2}-{\bar{\sigma }}_{x}^{2}. \end{array}$$

It is easy to verify that $\text{E}\left[{\overset{ˇ}{\sigma }}_{x}^{2}\right]=\sigma_{x}^{2}$ and thus ${\overset{ˇ}{\sigma }}_{x}^{2}$ provides an unbiased estimate for the spread $\sigma_{x}^{2}$. However, since the estimate has to be non-negative we use a modified estimate:

$$\begin{array}{c} {\hat{\sigma }}_{x}^{2}=\left\{\begin{array}{cc} \frac{1}{K-1}\sum_{i=1}^{K}{\left(x_{i}^{'}-\bar{x}\right)}^{2}-{\bar{\sigma }}_{x}^{2}\; & \text{if}\;\;\frac{1}{K-1}\sum_{i=1}^{K}{\left(x_{i}^{'}-\bar{x}\right)}^{2}>{\bar{\sigma }}_{x}^{2}\\ \frac{1}{K-1}\sum_{i=1}^{K}{\left(x_{i}^{'}-\bar{x}\right)}^{2} & \text{else}. \end{array}\right. \end{array}$$ (8)

Finally we estimate the true *x*-coordinates of the points in the cluster as

$$\begin{array}{c} {\hat{x}}_{i}=\bar{x}+\frac{{\hat{\sigma }}_{x}}{{\left({\hat{\sigma }}_{x}^{2}+\sigma_{x,i}^{2}\right)}^{\frac{1}{2}}}\left(x_{i}^{'}-\bar{x}\right). \end{array}$$ (9)

The justification for using this estimate is that the expectation of the pairwise squared distances between the estimated points remains the same as that of the true points (details in S1 Text). Therefore, quantification measures based on distance metrics, computed based on the estimated true points, must also be accurate.

Finally, combining the estimates of both coordinates, we get estimates ${\hat{X}}_{i}$ for the location of each molecule within the cluster. We separately apply this procedure to all observed clusters. Finally, we estimate the correct *K*-function using the estimated points as

$$\begin{array}{c} K\left(r\right)=\frac{\sum_{i\neq j}I\{∥{\hat{X}}_{i}-{\hat{X}}_{j}∥\leq r\}/N}{N/A}. \end{array}$$

This procedure can be further refined by incorporating the differences in the variances of different points while estimating the x-coordinate of the cluster centre in Eq. (6).

### Validation and simulation details

The demonstrative examples and *in silico* validation were done on “true points” (all label locations known with perfect accuracy), obtained either by simulation of point patterns or by setting the localizations obtained from a PALM experiment as the true points (Fig. 1a). The latter is useful in that it provides a point pattern that more closely reflects a molecular spatial distribution in practice.

The clustered point patterns used for the simulations were obtained in the following way. Gaussian clusters with a set standard deviation *σ* were generated in a 2*μm*x2*μm* area for different cluster densities (mean number of points that are clustered per unit area, values tested: 1000,100 and 10 *μm* ^−2^) and mean number of points per cluster (100, 30 and 10). A random point pattern is superimposed on the clustered points as background(“monomer fraction” of 30%). For S3 Fig., the cluster size *σ* was varied between 10nm and 100nm, and only the conditions of 10 and 100 molecules per cluster are displayed. For S4 Fig., the cluster size (*σ*) was 5nm and 10nm, the number of molecules per cluster 2 and 4, and the density 10 and 1000 *μm* ^−2^. Examples of point patterns used for simulations are shown in S8 Fig. The “true” *K*(*r*) or *L*(*r*) − *r* functions are then estimated using Eq. (3) and Eq. (2) (S9 Fig.).

Multiple realizations of the observed point patterns are then obtained by Monte Carlo simulations, based on the true points and the application of the effects of localization uncertainty and/or limited detection efficiency. For the observed points, the effect of subsampling corresponding to limited detection efficiency is added by generating a pseudo random number for each true point, sampled from a standard uniform distribution in (0,1), and the point is considered “sampled” if the sampling ratio is greater than this value. The effect of localization uncertainty is added by sampling from a Gaussian uncertainty distribution centered at each true location, with the standard deviation sampled from a distribution corresponding to an exponential photon count model [12] (S10 Fig.). The *K*(*r*) or *L*(*r*) − *r* functions are then estimated. The estimations provided by the analytical methods presented are also computed on the basis of true points and the same error parameters used in simulations.

The relative mean squared error (MSE) or the relative standard deviation (*σ*) is then computed by dividing with the true *K*(*r*) or *L*(*r*) − *r* values. The results are either averaged over *r* (S2 Fig.) or only the value at the true cluster radius (estimated from the maxima of *L*_*true*_(*r*) − *r* function) is used (other figures), and are then averaged over the different realizations of true point patterns for the same cluster condition (if the true points were obtained through simulations).

## Supporting Information

S1 Fig Propagation of localization error in cluster analysis with Ripleys *L*(*r*) − *r* function. *L*(*r*) − *r* curves are plotted for a set of points obtained from a PALM experiment (shown in Fig. 1 of main text), called true points, setting them as the true locations of molecules; and also for 1000 realizations of localizations estimated from these true points with a given estimation uncertainty model. (a) *L*(*r*) − *r* curves corresponding to the true points (dark green) and 1000 realizations of the estimated localizations with different localization precisions (red: mean precision 10nm. orange: 20nm. green: 30nm). Details on the models for localization uncertainty and the distribution of localization precision can be found in Methods. (b) The cluster radius estimated from the maxima of different *L*(*r*) − *r* curves. It can be seen that the *L*(*r*) − *r* curves and the cluster radius corresponding to the estimated localizations is far off from the ones corresponding to true points, and the difference increases with worse localization precision. Also see S7 Fig., for a method to predict the mean *L*(*r*) − *r* curves given the true points and localization precisions.
(TIF)

S2 Fig Validation of analytical method for estimating the variance in *K*-function due to subsampling. Comparison of mean relative standard deviation in *K*-function due to subsampling (Relative $\sigma =\frac{\sigma_{K,subsampled}}{K_{true}}$), estimated by the theoretical method (section Exact computation of variance of *K*-function due to random subsampling in Methods), to that estimated from simulations (100 realizations of subsampling per point pattern). The comparison is done for different cluster conditions (denoted by different symbols) and subsampling ratios (colors), after averaging over *r*. It can be seen that the relation follows a linear pattern *y* = *x* (note: plot is in log scale), i.e. the theoretical predictions match those from simulations. Details of the simulations can be found in Methods. The averaging was done over 10 point patterns per cluster condition.
(TIFF)

S3 Fig Characterization of relative standard deviation in *L*(*r*) − *r* due to random subsampling, at *r* = *r*_*true*_. Relative $\sigma =\frac{\sigma_{subsampled}}{L_{true}(r)-r}$, *r* = *r*_*true*_ is the true cluster radius estimated from *L*_*true*_(*r*) − *r*. The comparison is on the basis for simulated points, with different cluster conditions (solid & broken lines: number of molecules per cluster; markers: density; color: cluster size (SD of Gaussian)) and subsampling ratios (x axis).
For all the clustering conditions presented, for 60% sampling, the relative *σ* remained less than .25, with most obtaining a value less than .15. For a broad range of clustering conditions, the value remained less than .1 even for 20% sampling. It can be observed that the relative *σ* increases with increasing cluster radius, other cluster conditions remaining same (that is, when clusters become less dense). Details of the cluster simulations can be found in Methods. The averaging was done over 10 point patterns per cluster condition.
(TIFF)

S4 Fig Characterization of relative standard deviation in *L*(*r*) − *r* due to random subsampling, at *r* = *r*_*true*_, in the case of dimers and tetramers. Relative $\sigma =\frac{\sigma_{subsampled}}{L_{true}(r)-r}$. For sampling ratios of 60% or above, the relative *σ* remained less than .15 for the clustering conditions tested. Details of the cluster simulations can be found in Methods. The averaging was done over 10 point patterns per cluster condition.
(TIFF)

S5 Fig Application of reconstruction method on point locations from a PALM experiment as the true locations, and errors added artificially. The point pattern used as true points is the same as the one in Fig. 1 and S1 Fig. and the errors applied to get observed points are: mean localization precision 10nm, 20nm, 30nm and 50% subsampling. The cluster radius corresponding to true, observed and reconstructed points, estimated from the maxima of *L*(*r*) − *r* curves are displayed here. It can be seen that the ones corresponding to the reconstructed points are much closer to the true ones and its accuracy decreases with worse localization precision. The validation is important since it involves 1) a point pattern distributed as the one observed in a real PALM experiment rather than Gaussian clusters used in simulations, 2) the reconstruction also involves clustering, done by means of a clustering algorithm DBSCAN, rather than the perfect clustering (due to prior knowledge) used in simulations. A discussion on the importance of accurate clustering can be found in S1 Text.
(TIF)

S6 Fig Example application of reconstruction method on previously published cluster analysis. The method is applied on a previously published cluster analysis data, to note the extent of deviation between the *L*(*r*) − *r* curves corresponding to the observed and the reconstructed data. The data is from the same experiment as that shown in Fig. 1, published in Fig. 2 of Scarselli et al [13]. From the raw PALM localizations, localizations that appear multiple times are lumped together by setting a temporal threshold of 100 frames (of 10ms exposure). The localization precisions were computed with the expression provided in [20], for least squares fitting. All points that were localized with a precision that is better than 35nm was used for analysis, whether they are appearing in clusters or not. Clustering was performed for the reconstruction method using DBSCAN algorithm, with parameters *ϵ* = 20*nm* and *minpts* = 3. The curve corresponding to the reconstructed points (estimated true points, green) deviates from that corresponding to the observed points (red).
(TIF)

S7 Fig Demonstration of the method for the computation of *L*-function in the presence of localization uncertainty. A Gaussian clustered point pattern (20 points per *μm* ^2^, 10 points per cluster, 30nm average cluster standard deviation) is observed with localization precision distributions similar to the ones shown in S10 Fig., with mean precisions 10nm, 20nm and 30nm respectively. The *L*(*r*) − *r* curves are plotted for the true points (dark green), and that for the mean corresponding to 1000 realizations of the observed points, sampling from the uncertainty distribution (blue broken lines). The approximate mean *L*(*r*) − *r* as predicted by the presented method for the three cases is also plotted, computed from the exact *K*-function obtained, and can be seen as coinciding with those from the simulations.
(TIF)

S8 Fig Example point patterns used in simulations. Rows (top to bottom): Density of 1000 per *μm* ^2^ (a, b, c), 100 per *μm* ^2^ (d, e, f) and 10 per *μm* ^2^ (g, h). Columns (left to right): Molecules per cluster: 100 (a, d), 30 (b, e, g) and 10 (c, f, h). Example *L*(*r*) − *r* functions estimated can be found in S9 Fig.
(TIF)

S9 Fig Example *L*(*r*) − *r* curves used in simulations. (a) Mean *L*(*r*) − *r* functions (true, observed and reconstructed) corresponding to a particular cluster condition (density: 1000 per *μm* ^2^, 30 molecules per cluster). It can be noted that, in this case, the curves corresponding to the observed points with the same precision coincide approximately despite different subsampling ratios, as predicted by the invariance property of *L*(*r*) − *r* to random subsampling. (b) Example *L*(*r*) − *r* curves (true, observed) for the same number of molecules per cluster (10), but different density (1000 and 100 per *μm* ^2^). The relative effects of subsampling and localization uncertainty on the L(r)-r estimates can be observed. Also, even though the absolute variance (*σ*_*observed*_) is higher for the point pattern with lower density (100 per *μm* ^2^) for the same error conditions, the relative variation ($\frac{\sigma_{observed}}{L_{true}(r)-r}$) can be higher for the case of higher density (1000 per *μm* ^2^), as found in other figures.
(TIF)

S10 Fig Distributions of localization precision used in the simulations. The distributions shown hereby, based on an exponential model for the photons collected, are the ones that were used for Figure 5a, b and c respectively.
(TIF)

S1 Text Supporting information, with details of analytical derivation and discussion of effect of clustering errors on reconstruction. (PDF)

S1 Code MATLAB function to estimate the mean and the standard deviation of the subsampled *L*(*r*) − *r* given true points. (M)

S2 Code MATLAB function implementing the reconstruction method. (M)
