# Supplementary figures and images for: Accounting for Limited Detection Efficiency and Localization Precision in Cluster Analysis in Single Molecule Localization Microscopy
Source: PLoS One. 2015 Mar 20;10(3):e0118767. doi: 10.1371/journal.pone.0118767 (PMC4368834; doi:10.1371/journal.pone.0118767)

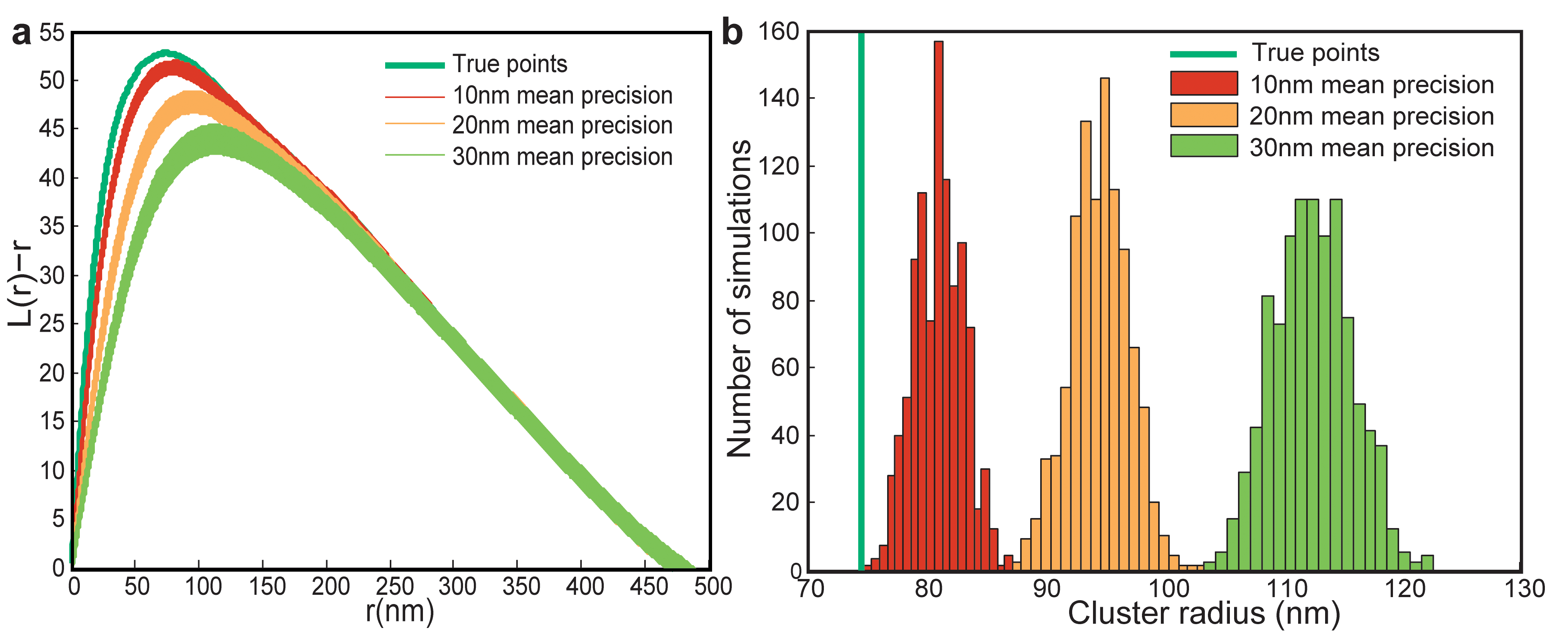

Supplement: S1 Fig — L(r) − r curves are plotted for a set of points obtained from a PALM experiment (shown in Fig. 1 of main text), called true points, setting them as the true locations of molecules; and also for 1000 realizations of localizations estimated from these true points with a given estimation uncertainty model. (a) L(r) − r curves corresponding to the true points (dark green) and 1000 realizations of the estimated localizations with different localization precisions (red: mean precision 10nm. orange: 20nm. green: 30nm). Details on the models for localization uncertainty and the distribution of localization precision can be found in Methods. (b) The cluster radius estimated from the maxima of different L(r) − r curves. It can be seen that the L(r) − r curves and the cluster radius corresponding to the estimated localizations is far off from the ones corresponding to true points, and the difference increases with worse localization precision. Also see S7 Fig., for a method to predict the mean L(r) − r curves given the true points and localization precisions. (TIF) [file pone.0118767.s001.tif]

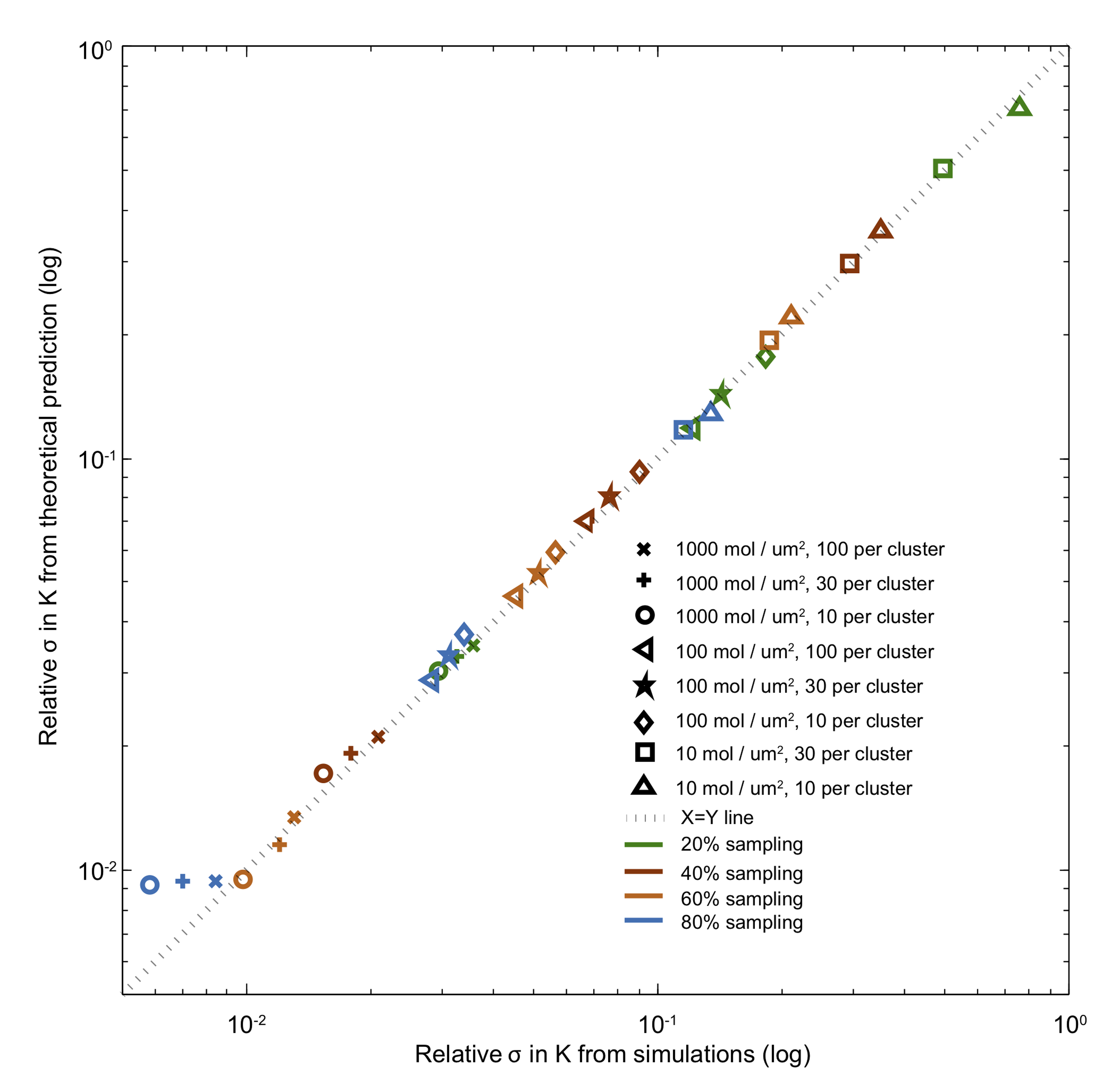

Supplement: S2 Fig — Comparison of mean relative standard deviation in K-function due to subsampling (Relative σ=σK,subsampledKtrue), estimated by the theoretical method (section Exact computation of variance of K-function due to random subsampling in Methods), to that estimated from simulations (100 realizations of subsampling per point pattern). The comparison is done for different cluster conditions (denoted by different symbols) and subsampling ratios (colors), after averaging over r. It can be seen that the relation follows a linear pattern y = x (note: plot is in log scale), i.e. the theoretical predictions match those from simulations. Details of the simulations can be found in Methods. The averaging was done over 10 point patterns per cluster condition. (TIFF) [file pone.0118767.s002.tiff]

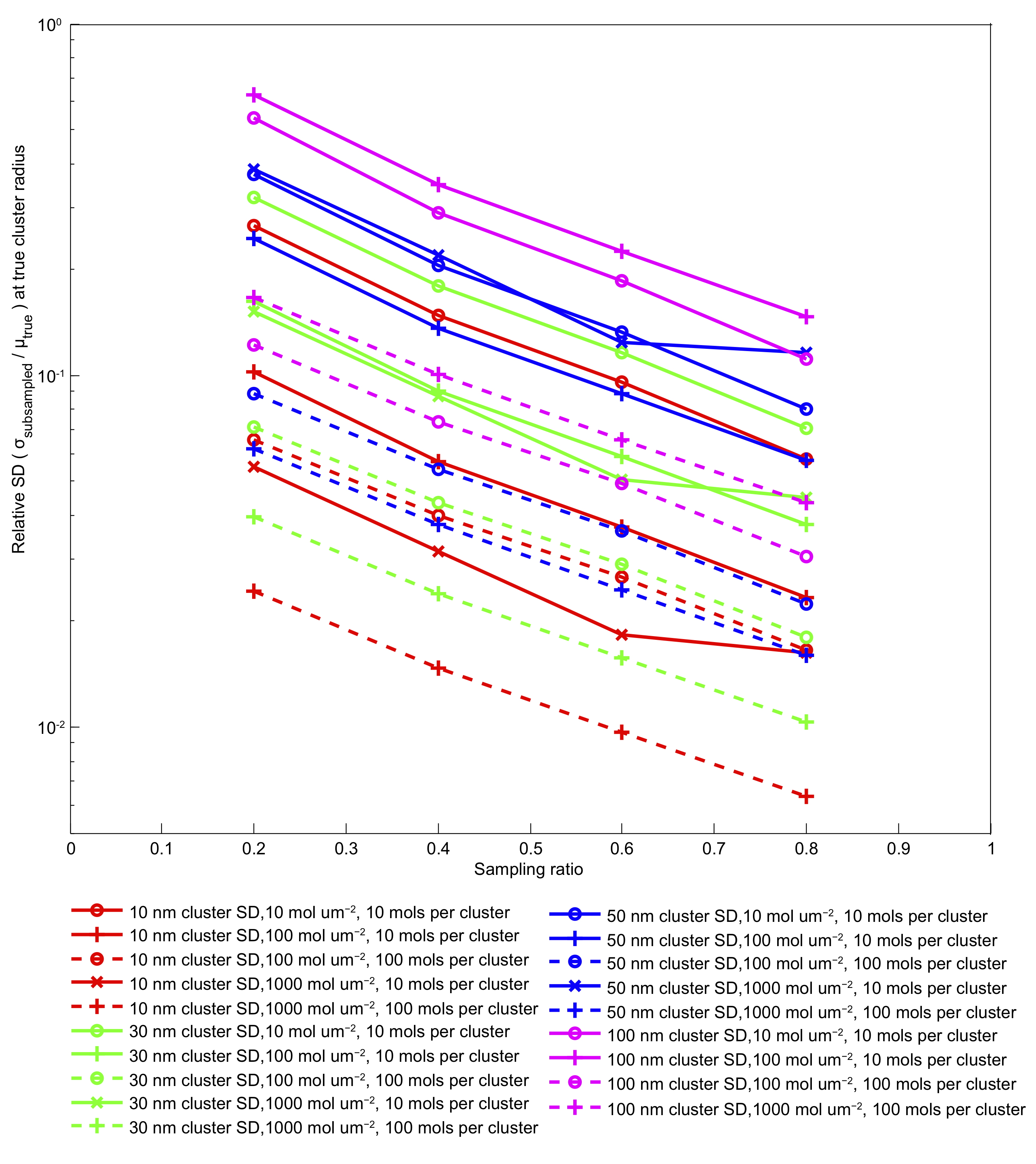

Supplement: S3 Fig — Relative σ=σsubsampledLtrue(r)−r, r = r true is the true cluster radius estimated from L true(r) − r. The comparison is on the basis for simulated points, with different cluster conditions (solid & broken lines: number of molecules per cluster; markers: density; color: cluster size (SD of Gaussian)) and subsampling ratios (x axis). For all the clustering conditions presented, for 60% sampling, the relative σ remained less than .25, with most obtaining a value less than .15. For a broad range of clustering conditions, the value remained less than .1 even for 20% sampling. It can be observed that the relative σ increases with increasing cluster radius, other cluster conditions remaining same (that is, when clusters become less dense). Details of the cluster simulations can be found in Methods. The averaging was done over 10 point patterns per cluster condition. (TIFF) [file pone.0118767.s003.tiff]

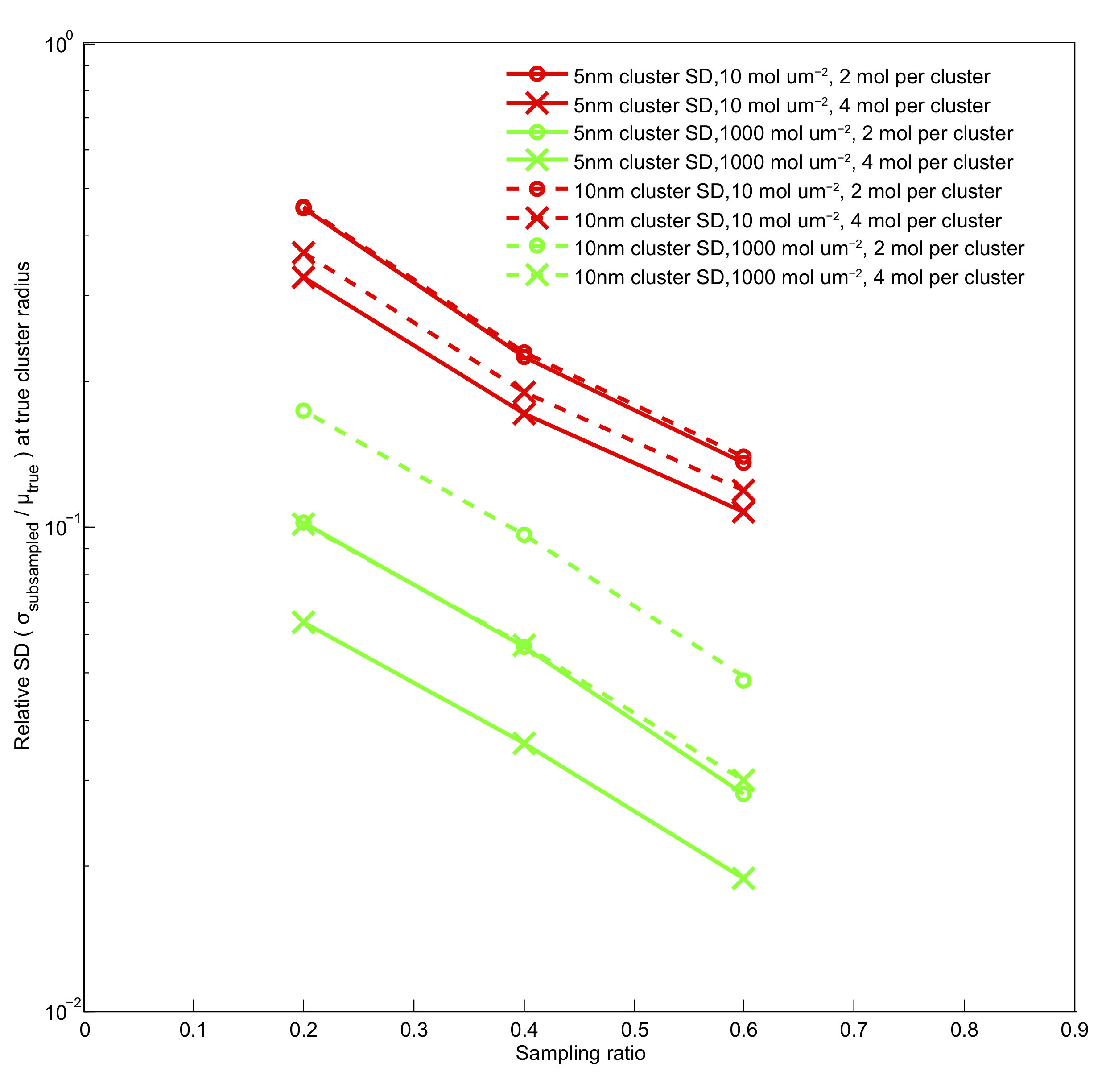

Supplement: S4 Fig — Relative σ=σsubsampledLtrue(r)−r. For sampling ratios of 60% or above, the relative σ remained less than .15 for the clustering conditions tested. Details of the cluster simulations can be found in Methods. The averaging was done over 10 point patterns per cluster condition. (TIFF) [file pone.0118767.s004.tiff]

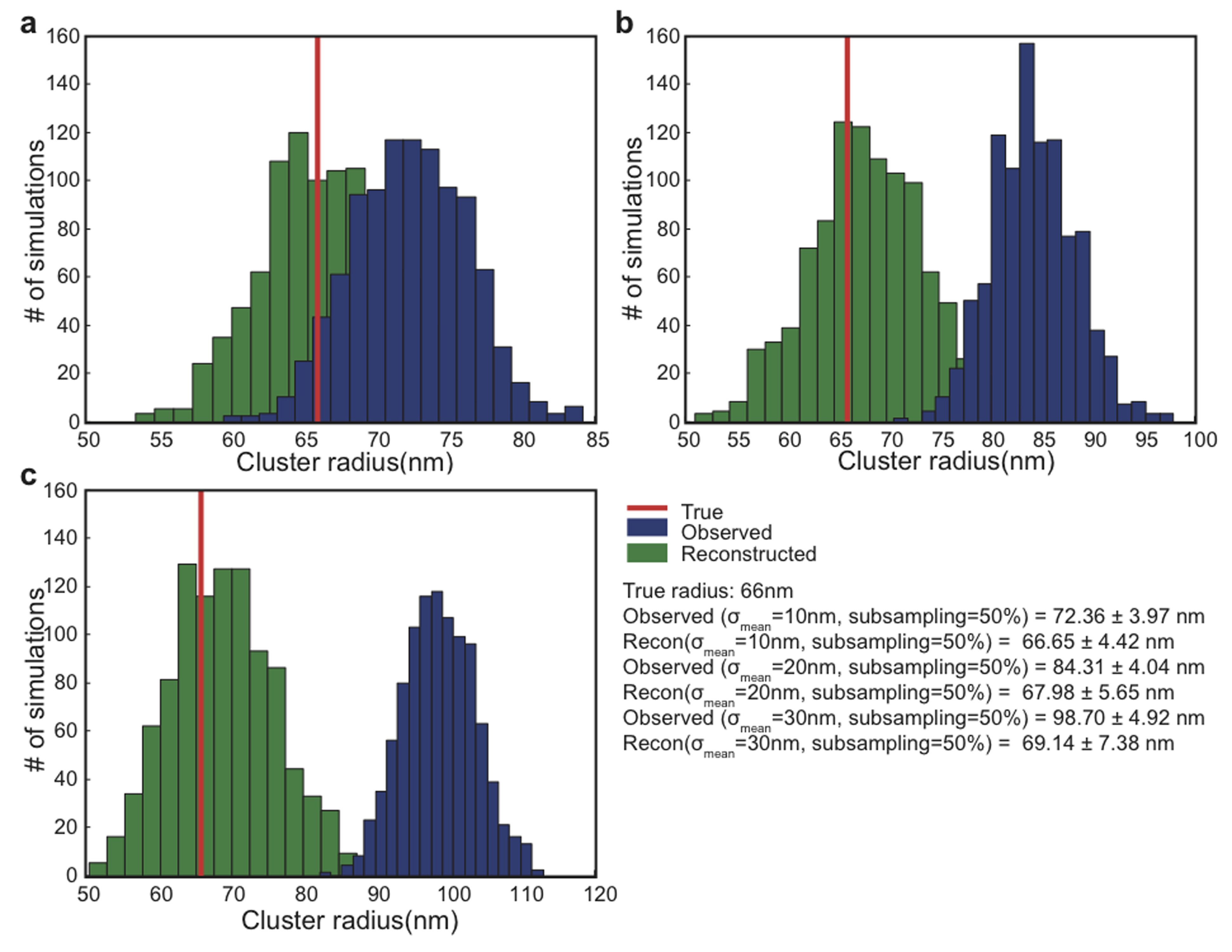

Supplement: S5 Fig — The point pattern used as true points is the same as the one in Fig. 1 and S1 Fig. and the errors applied to get observed points are: mean localization precision 10nm, 20nm, 30nm and 50% subsampling. The cluster radius corresponding to true, observed and reconstructed points, estimated from the maxima of L(r) − r curves are displayed here. It can be seen that the ones corresponding to the reconstructed points are much closer to the true ones and its accuracy decreases with worse localization precision. The validation is important since it involves 1) a point pattern distributed as the one observed in a real PALM experiment rather than Gaussian clusters used in simulations, 2) the reconstruction also involves clustering, done by means of a clustering algorithm DBSCAN, rather than the perfect clustering (due to prior knowledge) used in simulations. A discussion on the importance of accurate clustering can be found in S1 Text. (TIF) [file pone.0118767.s005.tif]

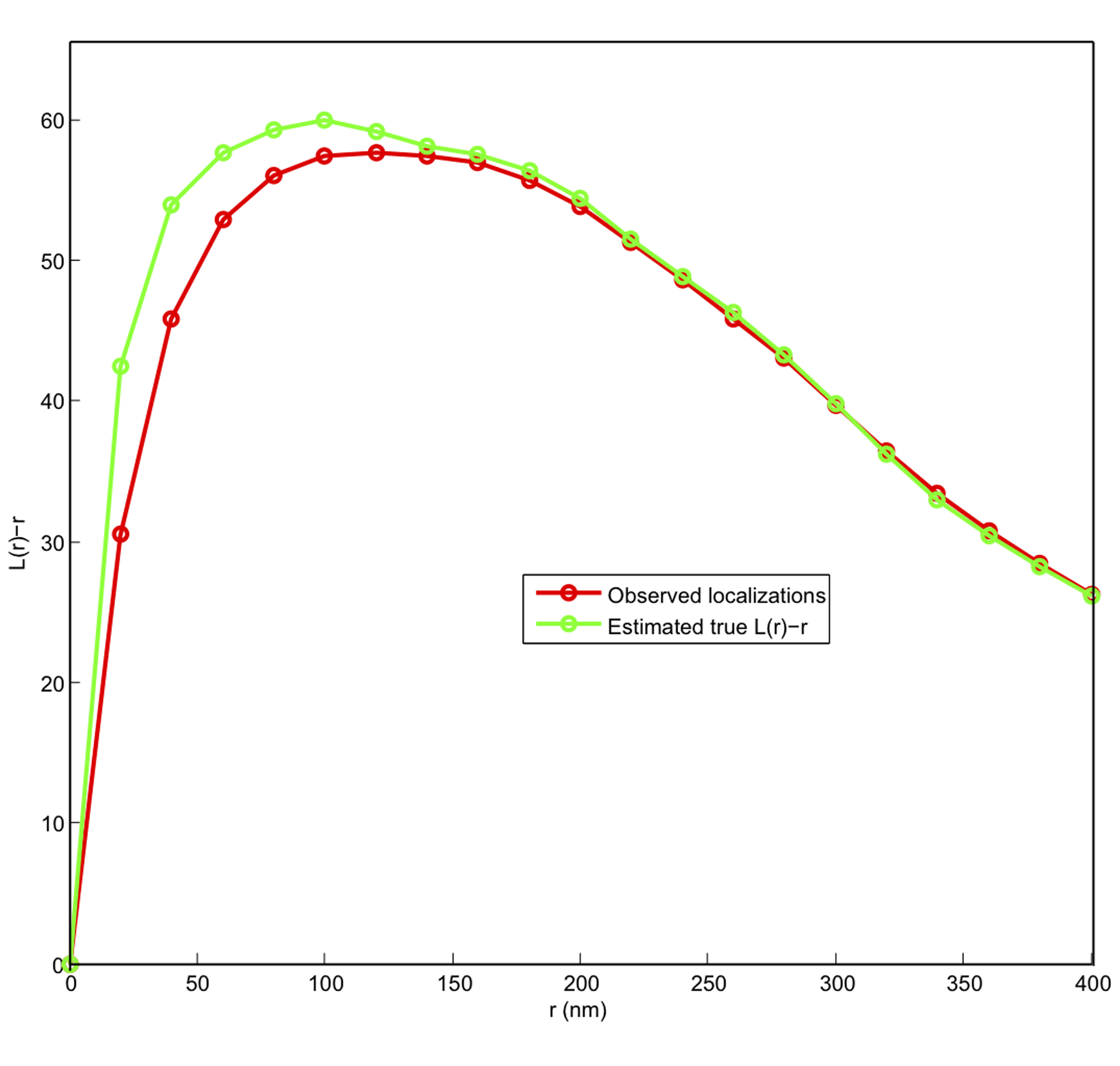

Supplement: S6 Fig — The method is applied on a previously published cluster analysis data, to note the extent of deviation between the L(r) − r curves corresponding to the observed and the reconstructed data. The data is from the same experiment as that shown in Fig. 1, published in Fig. 2 of Scarselli et al [13]. From the raw PALM localizations, localizations that appear multiple times are lumped together by setting a temporal threshold of 100 frames (of 10ms exposure). The localization precisions were computed with the expression provided in [20], for least squares fitting. All points that were localized with a precision that is better than 35nm was used for analysis, whether they are appearing in clusters or not. Clustering was performed for the reconstruction method using DBSCAN algorithm, with parameters ϵ = 20nm and minpts = 3. The curve corresponding to the reconstructed points (estimated true points, green) deviates from that corresponding to the observed points (red). (TIF) [file pone.0118767.s006.tif]

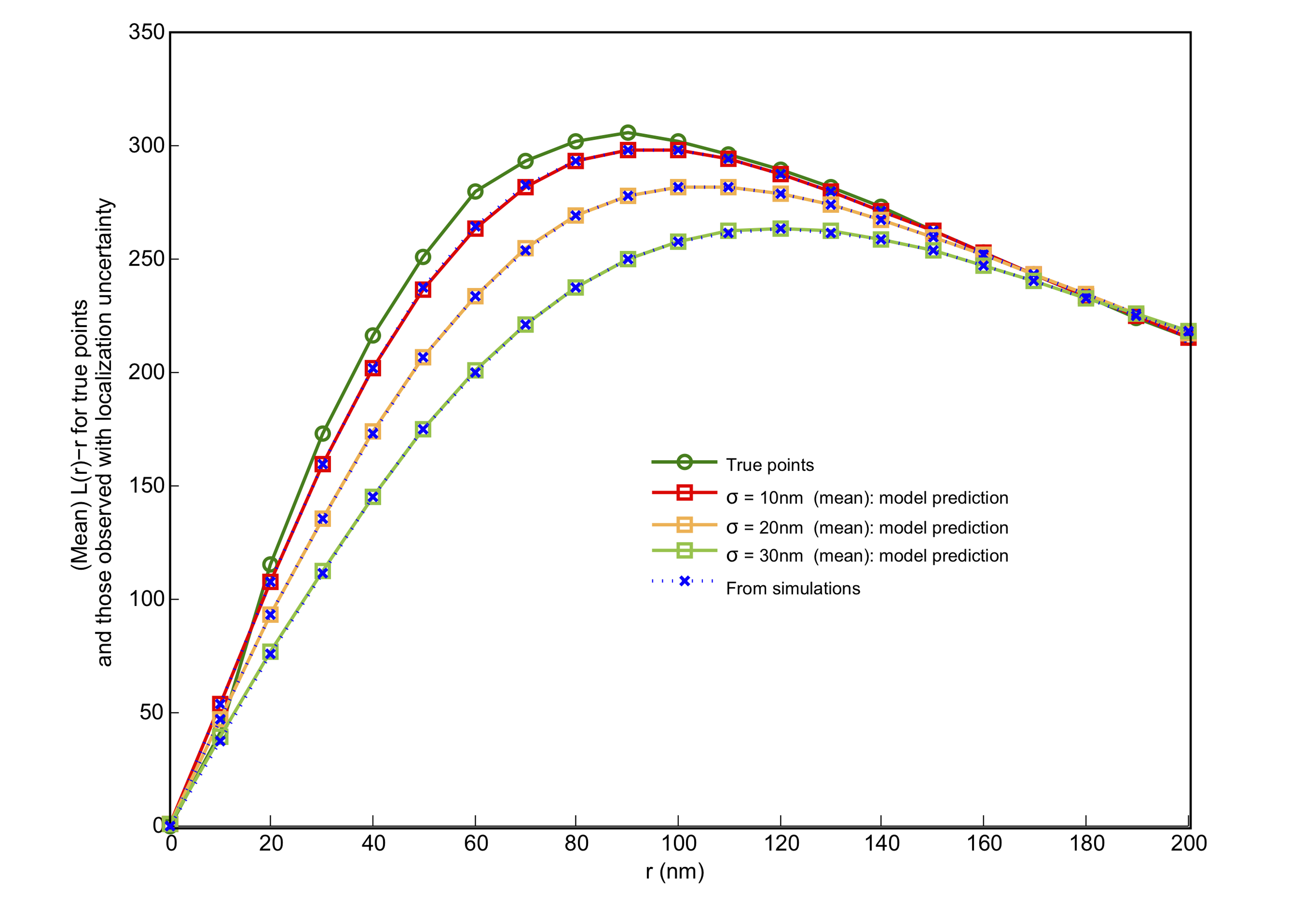

Supplement: S7 Fig — A Gaussian clustered point pattern (20 points per μm 2, 10 points per cluster, 30nm average cluster standard deviation) is observed with localization precision distributions similar to the ones shown in S10 Fig., with mean precisions 10nm, 20nm and 30nm respectively. The L(r) − r curves are plotted for the true points (dark green), and that for the mean corresponding to 1000 realizations of the observed points, sampling from the uncertainty distribution (blue broken lines). The approximate mean L(r) − r as predicted by the presented method for the three cases is also plotted, computed from the exact K-function obtained, and can be seen as coinciding with those from the simulations. (TIF) [file pone.0118767.s007.tif]

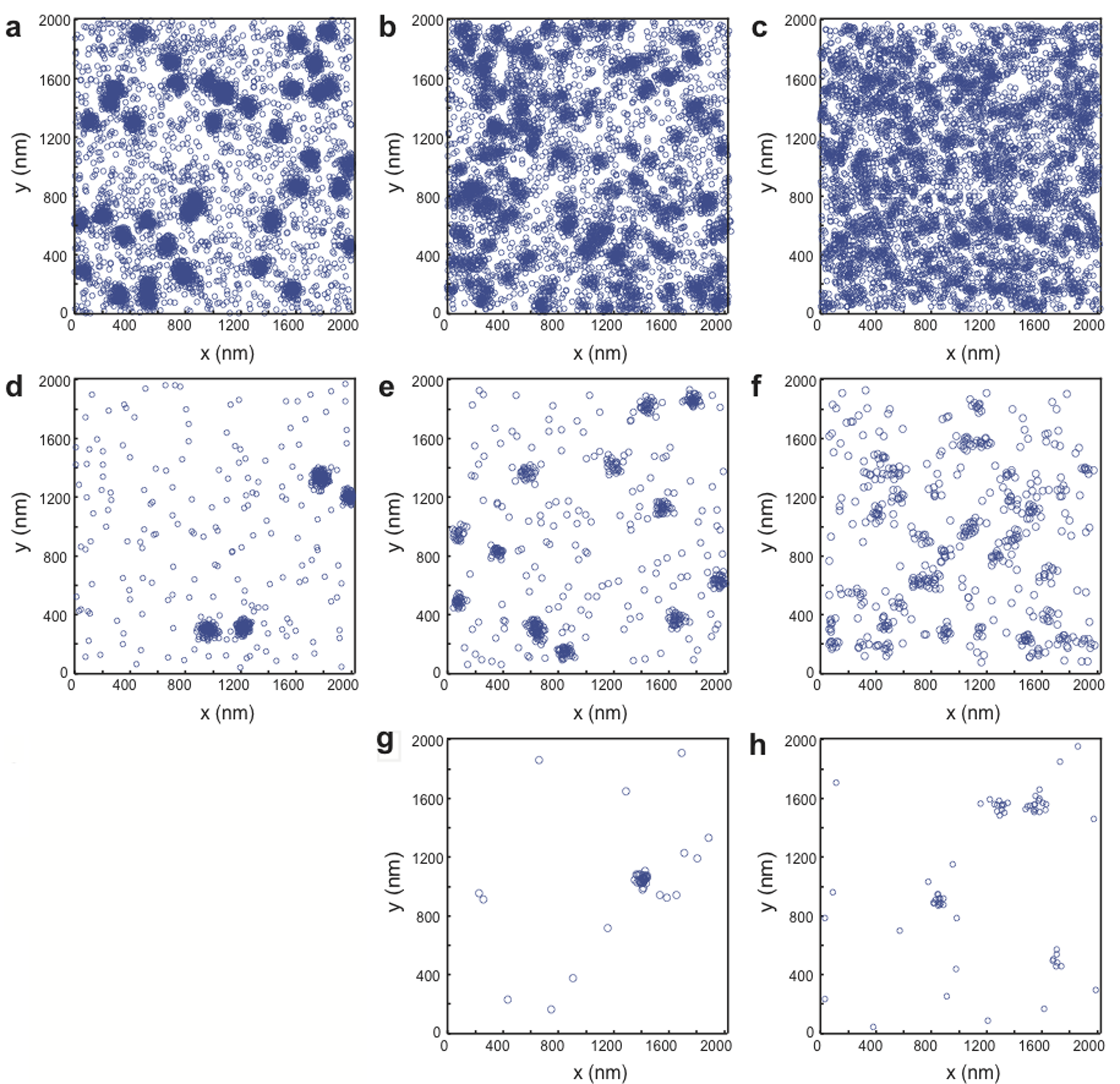

Supplement: S8 Fig — Rows (top to bottom): Density of 1000 per μm 2 (a, b, c), 100 per μm 2 (d, e, f) and 10 per μm 2 (g, h). Columns (left to right): Molecules per cluster: 100 (a, d), 30 (b, e, g) and 10 (c, f, h). Example L(r) − r functions estimated can be found in S9 Fig. (TIF) [file pone.0118767.s008.tif]

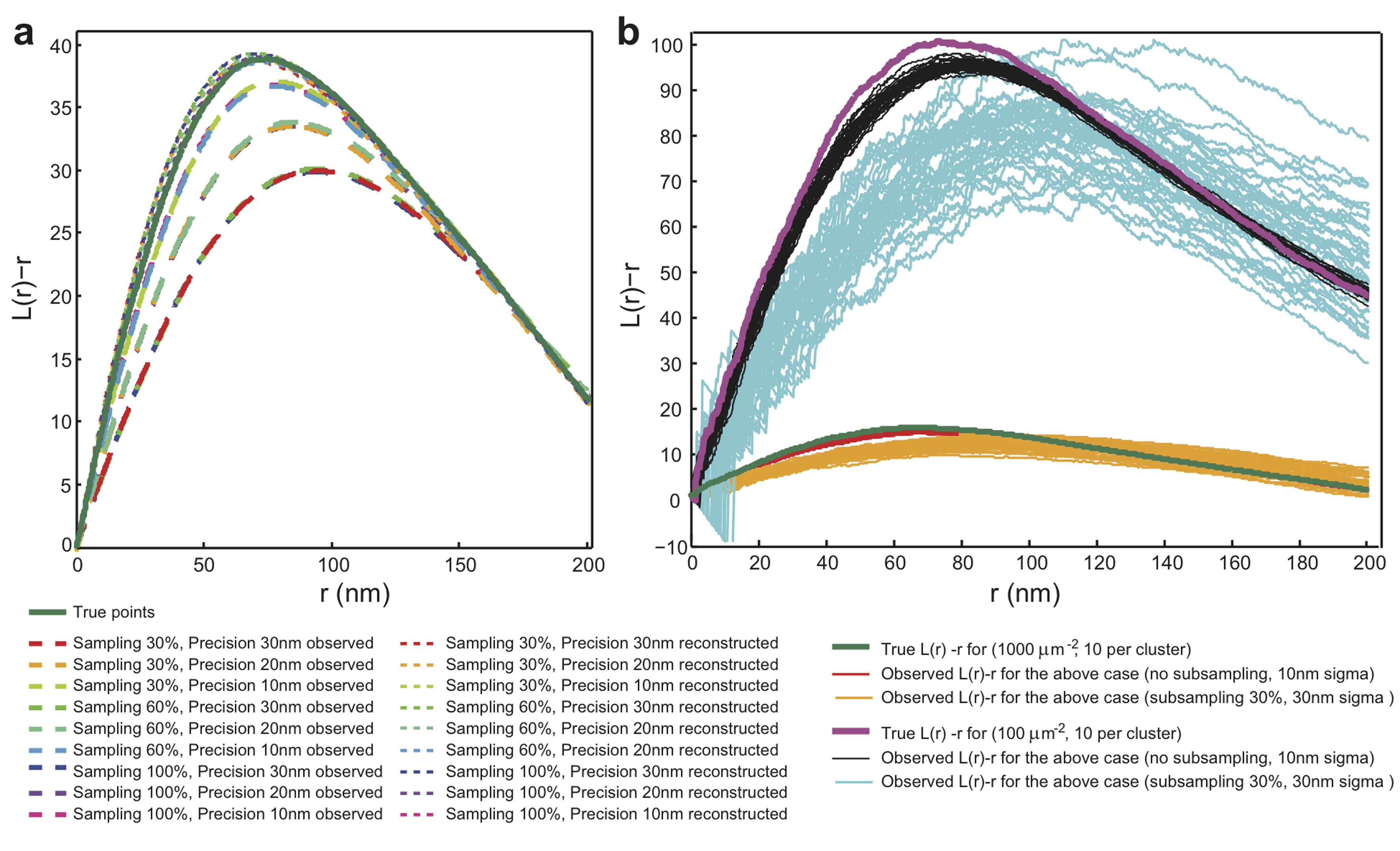

Supplement: S9 Fig — (a) Mean L(r) − r functions (true, observed and reconstructed) corresponding to a particular cluster condition (density: 1000 per μm 2, 30 molecules per cluster). It can be noted that, in this case, the curves corresponding to the observed points with the same precision coincide approximately despite different subsampling ratios, as predicted by the invariance property of L(r) − r to random subsampling. (b) Example L(r) − r curves (true, observed) for the same number of molecules per cluster (10), but different density (1000 and 100 per μm 2). The relative effects of subsampling and localization uncertainty on the L(r)-r estimates can be observed. Also, even though the absolute variance (σ observed) is higher for the point pattern with lower density (100 per μm 2) for the same error conditions, the relative variation (σobservedLtrue(r)−r) can be higher for the case of higher density (1000 per μm 2), as found in other figures. (TIF) [file pone.0118767.s009.tif]

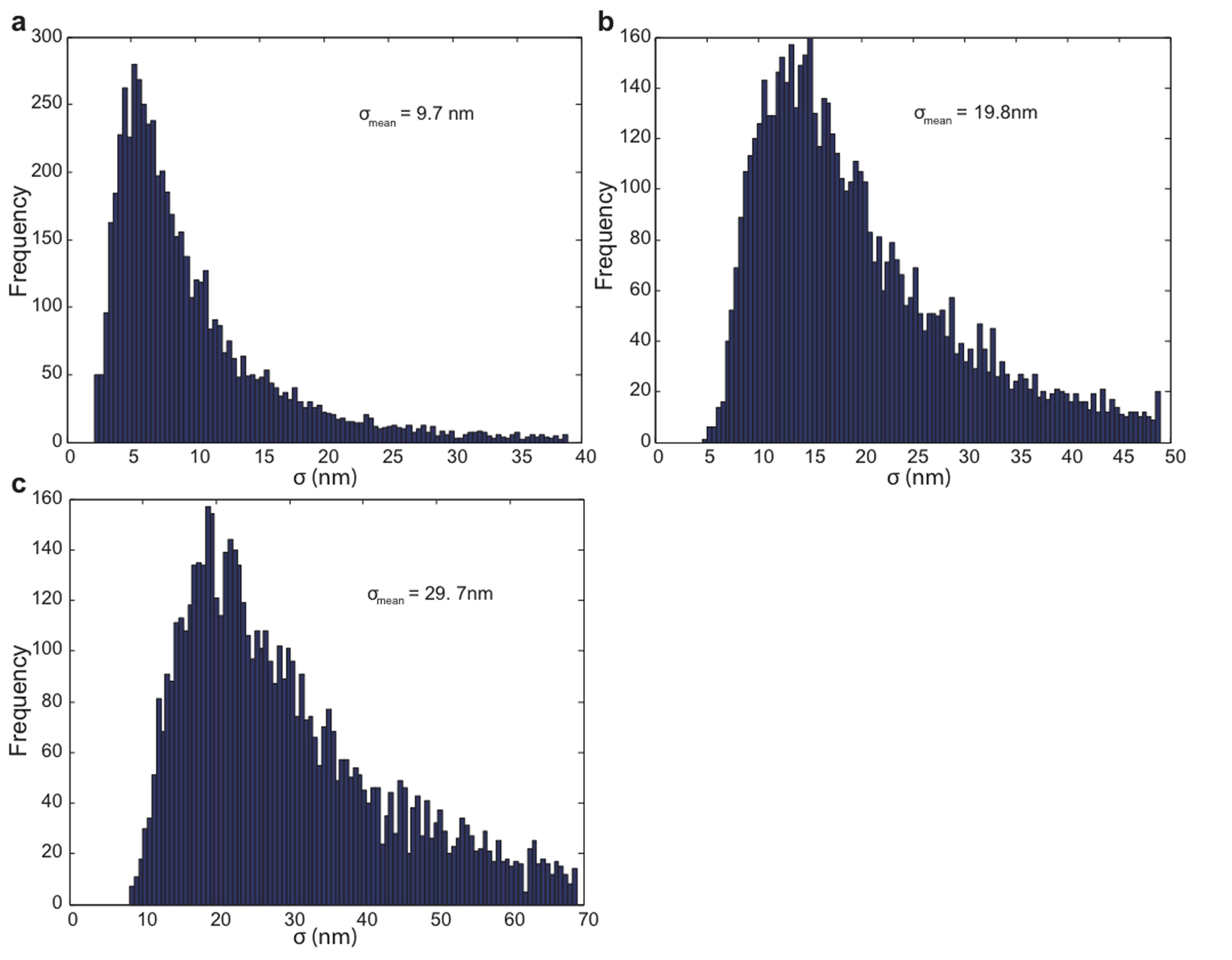

Supplement: S10 Fig — The distributions shown hereby, based on an exponential model for the photons collected, are the ones that were used for Figure 5a, b and c respectively. (TIF) [file pone.0118767.s010.tif]
