# Supplementary material for: Accounting for Limited Detection Efficiency and Localization Precision in Cluster Analysis in Single Molecule Localization Microscopy
Source: PLoS One. 2015 Mar 20;10(3):e0118767. doi: 10.1371/journal.pone.0118767 (PMC4368834; doi:10.1371/journal.pone.0118767)
Supplement: S1 Text — (PDF) [file pone.0118767.s011.pdf]

Supporting Information

Exact computation of variance of K-function due to random subsampling

The randomness in the estimate of $K(r)$ due to subsampling arises due to the Bernoulli random variables $B_1, B_2, \ldots, B_N$. The mean and variance of $K(r)$ can be estimated using the first and second moments of $K(r)$.

The first moment is

$$\mathsf{E}[K(r)] = \sum_{i \neq j} \mathsf{E}\left[\frac{B_i B_j}{(\sum_{i=1}^N B_i)^2}\right] \mathcal{I}\{\|X_i - X_j\| \leq r\} A \tag{1}$$

and the second moment is

$$\mathsf{E}[K(r)^2] = \sum_{i \neq j} \sum_{k \neq \ell} \mathsf{E}\left[\frac{B_i B_j B_k B_\ell}{(\sum_{i=1}^N B_i)^4}\right] \mathcal{I}\{\|X_i - X_j\| \leq r\} \mathcal{I}\{\|X_k - X_\ell\| \leq r\} A^2. \tag{2}$$

Since B_i are i.i.d. and $B_i^2 = B_i$, in order to evaluate the expectations in (2) and (2), it is sufficient to compute $\mathsf{E}\left[\frac{B_1 B_2}{(\sum_{i=1}^N B_i)^2}\right]$, $\mathsf{E}\left[\frac{B_1 B_2 B_3}{(\sum_{i=1}^N B_i)^2}\right]$ and $\mathsf{E}\left[\frac{B_1 B_2 B_3 B_4}{(\sum_{i=1}^N B_i)^2}\right]$.

Let $\alpha = \mathsf{E}[B_1]$. We have

$$\begin{aligned}
\mathsf{E}\left[\frac{\prod_{j=1}^K B_j}{(\sum_{i=1}^N B_i)^2}\right] &= \sum_{k=1}^N \Pr\left\{\sum_{i=1}^N B_i = k\right\} \Pr\left\{\prod_{i=1}^K B_i = 1 | \sum_{i=1}^N B_i = k\right\} \frac{1}{k^2} \\
&= \sum_{k=1}^N \Pr\left\{\sum_{i=1}^N B_i = k\right\} \frac{\Pr\left\{\sum_{i=K+1}^N B_i = k-K\right\} \Pr\left\{\prod_{i=1}^K B_i = 1\right\}}{\Pr\left\{\sum_{i=1}^N B_i = k\right\}} \frac{1}{k^2} \\
&= \sum_{k=1}^N \Pr\left\{\sum_{i=K+1}^N B_i = k-K\right\} \Pr\left\{\prod_{i=1}^K B_i = 1\right\} \frac{1}{k^2} \\
&= \sum_{k=1}^N \binom{N-K}{k-K} \alpha^{k-K} (1-\alpha)^{N-k} \alpha^K \frac{1}{k^2} \\
&= \sum_{k=1}^N \binom{N-K}{k-K} \alpha^k (1-\alpha)^{N-k} \frac{1}{k^2}.
\end{aligned}$$

The last expression can be directly computed or approximated with an integral.

Exact computation of K-function in the presence of localization uncertainty

For example, if W_i are assumed to be independently drawn zero mean Gaussian random vectors, the vector $X_i - X_j + W_i - W_j$ is a Gaussian random vector with mean $X_i - X_j$, and covariance equal to the sum of

the covariances of W_i and W_j, and hence $\|X_i - X_j + W_i - W_j\|^2$ is a non-central χ^2 random variable, with known distribution. The expected value of $K'(r)$ is given by

$$\begin{aligned} \mathsf{E}[K'(r)] &= \frac{\sum_{i\neq j} \mathsf{E}\left[\mathcal{I}\{\|X_i' - X_j'\| \leq r\}\right]/N}{N/A} \\ &= \frac{\sum_{i\neq j} \mathsf{E}\left[\mathcal{I}\{\|X_i - X_j + W_i - W_j\|^2 \leq r^2\}\right]/N}{N/A}. \end{aligned}$$

The expected value inside the summation is nothing but the complementary cumulative distribution function of a non-central χ^2 random variable, which is easily computed using the Marcum Q-function.

Justification for the choice of estimator of true locations

In the Methods section of main text, the following estimators are defined.

$$\hat{x}_i = \overline{x} + \frac{\hat{\sigma}_x}{(\hat{\sigma}_x^2 + \sigma_{x,i}^2)^{\frac{1}{2}}}(x_i' - \overline{x}). \tag{3}$$

$$\overline{x} = \frac{1}{K}\sum_{i=1}^{K} x_i', \tag{4}$$

$$\overline{\sigma}_x^2 = \frac{1}{K}\sum_{i=1}^{K} \sigma_{x,i}^2. \tag{5}$$

The estimate of (3) can be justified under the assumption that estimates of the x-coordinate of the cluster center in (4) and cluster spread of the x-coordinate in (5) are accurate. Let

$$\check{x}_i = \mu_x + \frac{\sigma_x}{(\sigma_x^2 + \sigma_{x,i}^2)^{\frac{1}{2}}}(x_i' - \mu_x)$$

denote the estimate of (3) when the cluster center and spread are accurate. It is easy to verify that

$$\mathsf{E}\left[(\check{x}_i - \check{x}_j)^2 - (x_i - x_j)^2\right] = 0.$$

This suggests that by using the estimates of (3), the estimate of the squared distance between any pair of points in the cluster is unbiased, and thus the K-function computed using distances between the estimated points is expected to be accurate. This is the main reason for using the estimate of (3). It is to be noted here that if one were interested in minimising the squared error $\mathsf{E}[\|\widehat{X}_i - X_i\|^2]$ in the position of each molecule, then one would use the MMSE estimator of

$$\overline{x} + \frac{\hat{\sigma}_x^2}{\hat{\sigma}_x^2 + \sigma_{x,i}^2}(x_i' - \overline{x})$$

in (3). However, it was observed that in practice this leads to a shrinking of the reconstructed clusters. The current estimator does not have this drawback and has the added advantage of accurately approximating distances between points in the cluster.

Effect of clustering of localizations on reconstruction

The reconstruction method presented in the paper works on a cluster-by-cluster basis, and therefore the SMLM localizations must be first preprocessed by means of clustering algorithms like DBSCAN [1] or others [2], before applying the method. The method presented assumes that the clustering errors are minimal. Example reconstructions after including clustering by DBSCAN is shown in Figure S5, which provided satisfactory results. The user is recommended to try out different clustering methods and parameters for a given dataset, so as to minimize the clustering errors. There are obvious limitations to this approach: in a case where the clusters are overlapping, it might be difficult for clustering algorithms to identify true clusters.

For the reconstruction method, since the X and Y coordinates are estimated separately, the method works best if the clusters are elliptical if not circular. As mentioned already, it is best if the clusters are well separated. Also, if a cluster with an arbitrarily complicated shape is clustered into multiple small symmetric clusters by the clustering algorithm, since the reconstruction method works on the basis of shrinking the clusters about a central point for each cluster, it might introduce artifacts, since each of the small clusters will be shrinked about their centers rather than the center of the true cluster. Therefore, the user must be careful to make sure that the clustering step does not introduce major errors or artifacts.

If the clusters are expected to have other specific parametric shapes, e.g., polygonal, helical etc., it might be possible to adapt the reconstruction method proposed in this paper to these alternate cluster shapes.

References

[1] Ester M, Kriegel HP, Sander J, Xu X (1996) A density-based algorithm for discovering clusters in large spatial databases with noise. In: Second International Conference on Knowledge Discovery and Data Mining. AAAI Press, pp. 226–231.

[2] Rodriguez A, Laio A (2014) Clustering by fast search and find of density peaks. Science 344: 1492-1496.

[3] Scarselli M, Annibale P, Radenovic A (2012) Cell type-specific 2-adrenergic receptor clusters identified using photoactivated localization microscopy are not lipid raft related, but depend on actin cytoskeleton integrity. J Biol Chem 287: 16768–16780.

[4] Mortensen KI, Churchman LS, Spudich JA, Flyvbjerg H (2010) Optimized localization analysis for single-molecule tracking and super-resolution microscopy. Nat Methods 7: 377-381.
